# Male Partners' Involvement in the Prevention of Mother-to-Child Transmission of HIV and Associated Factors in Arba Minch Town and Arba Minch Zuria Woreda, Southern Ethiopia

**DOI:** 10.1155/2015/763876

**Published:** 2015-06-03

**Authors:** Marelign Tilahun, Shikur Mohamed

**Affiliations:** ^1^Social and Public Health Unit, College of Health Sciences, Debre Tabor University, P.O. Box 272, Debre Tabor, Ethiopia; ^2^Department of Public Health, College of Medicine and Health Sciences, Arba Minch University, P.O. Box 21, Arba Minch, Ethiopia

## Abstract

*Background*. Male involvement is an important determinant of prevention of mother-to-child transmission of HIV. However, male involvement in prevention of mother-to-child transmission of HIV in Ethiopia is not well known. *Objectives*. To assess male partners involvement in prevention of mother-to-child transmission of HIV and associated factors in Arba Minch town and Arba Minch Zuria woreda. *Methods*. Community based study was conducted in Arba Minch town and Arba Minch Zuria district. Multistage sampling technique was used and data were collected using interviewer administered standard questionnaire. Multiple logistic regression analysis was used to determine the presence of statistically significant associations between the outcome variable and the independent variables. *Results*. The level of male involvement in PMTCT program in Arba Minch town and Zuria district was 53%. Several factors appear to contribute to male involvement in the PMTCT program including age, residence, education level, knowledge on HIV, knowledge on PMTCT, accessibility of health facility, having weak perception for male involvement in PMTCT, having perception of ANC attendance being females' responsibility, ever use of khat, and ever use of cigarette. *Conclusion*. Geographical accessibility of health facility and male's knowledge on PMTCT should be improved to increase their involvement in PMTCT.

## 1. Introduction

Mother-to-child HIV transmission of HIV remains a significant problem in the developing world despite the development and growing availability of effective prevention methods appropriate for resource-limited settings [1, 2]. Male partners play a role not only in women's risk of acquiring HIV but also in terms of her utilization of the PMTCT program: for the mother to test for HIV, for the mother to return for the result, for the couple to use condoms, for the mother to receive medication, and for her to follow the infant feeding advice given [1, 3–6]. In a previous study, it is observed that the involvement of male partners in antenatal VCT was associated with increased uptake of interventions to prevent vertical and sexual HIV transmission [4].

From other sub-Saharan African studies, the fear of a partner's negative reaction towards the mother testing for HIV and fear of disclosure of the test results have been found to be barriers to HIV testing for pregnant women in the PMTCT program [5, 7]. Other studies from sub-Saharan Africa found that couple HIV counseling appeared to improve the acceptability of HIV testing and uptake of ARV prophylaxis for PMTCT. Women who received couple counseling did not report an increased risk of adverse social events compared to individually counseled women [8–10]. Dropout among those who have discussed HIV testing with their partners was found to be low in Burkina Faso [11].

Studies in Kenya found that women accompanied by their partner for HIV testing were three times more likely to return for antiretroviral prophylaxis. Couple posttest counseling was also associated with an eightfold increase in postpartum follow-up, as well as greater antiretroviral utilization and formula feeding [4, 9]. In Uganda male involvement is associated with increased uptake of HIV testing and preventive interventions for vertical and sexual transmissions of HIV [12].

Male involvement is an important determinant factor of PMTCT service uptake. However no study was conducted on involvement of male partners in PMTCT programs in Ethiopia and particularly in the present study area. Therefore, this study was conducted to identify the determinant factors of male partner's involvement in the PMTCT program in Arba Minch town and Arba Minch Zuria district.

## 2. Methods

### 2.1. Study Design and Study Area

Community based study was conducted from February 2013 to September 2013. The study was conducted in Arba Minch town and Arba Minch Zuria district which is located about 505 km south of Addis Ababa, the capital of Ethiopia. There are three hospitals and sixty-eight health centers in Gamo-Gofa zone offering health care services for the total population.

### 2.2. Study Population

The study population was all male partners of reproductive age women (15–49 years) who give birth during the previous one year in Arba Minch town and Arba Minch Zuria woreda.

### 2.3. Sample Size Determination Method

Sample size of 720 was determined by the formula of two population proportion estimations using software Epi-info stat calc. with 95% confidence interval, power of 80%, odds ratio of 2.0, an estimated 26% male partners involvement in PMTCT [10], rural to urban ratio of 2 : 1, and a conservative design effect of 2.0 to accommodate for intracluster variability.

### 2.4. Sampling Procedure

Multistage sampling technique was used. There are 11 Kebeles in Arba Minch town and 29 Kebeles in Arba Minch Zuria woreda out of which three Kebeles in Arba Minch town and six Kebeles in Arba Minch Zuria woreda were selected by simple random sampling. Individual Kebele households having children who are less than one year were then selected using a systematic sampling technique.

### 2.5. Operational Definitions

*Male Involvement in PMTCT*. The level of male involvement in PMTCT was determined using the following six variables which were taken from previous literature [13]:

1. Did you know your wife's appointment for ANC the last time she was pregnant?
2. Did you discuss with your wife counseling and testing for HIV the last time she was pregnant?
3. Have you ever gone together with your wife to an ANC/PMTCT clinic?
4. Have you ever counselled and tested for HIV together with your wife at an ANC/PMTCT clinic?
5. Did you support your wife's antenatal visits financially?
6. Do you accept if health professionals inform you to use condom during the time of your wife's pregnancy?

The involvement score for each respondent could range from 0 = no involvement to 6 = involved in all 6 activities. A total score of 4–6 was considered as “involved in PMTCT” and 0–3 as “not involved in PMTCT” relative to this particular population and this score of measuring PMTCT involvement was taken from previous literature [13].

### 2.6. Data Collection and Quality Control

Data were collected using interviewer administered standard questionnaire. Individuals who have completed grade 12 were recruited as data collectors and were trained on the data collection procedures. Male involvement in PMTCT was considered as the outcome or dependent variable, while sociodemographic variables (age, residence, religion, ethnicity, income, occupation, and education level), residence, number of children, cohabitation duration, alcohol use, khat use, cigarette smoking, comprehensive knowledge on HIV/AIDS, perceived risk of HIV, knowledge on MTCT of HIV, knowledge on PMTCT of HIV, knowledge of services given at ANC clinic, and accessibility of health services were considered as independent variables.

### 2.7. Data Processing and Analysis

All returned questionnaires were checked for completeness and consistency of responses. After cleaning, data was coded and entered into and analyzed using SPSS for Windows version 20.0. Descriptive statistics such as frequencies, proportion, and cross tabulations were used to describe the study population in relation to relevant variables. Multiple logistic regression analysis was used to determine the strength of associations between the outcome variable and the independent variables.

### 2.8. Ethical Consideration

Ethical clearance was obtained from ethical review committee of Arba Minch University and permission was obtained from the respective Kebeles in Arba Minch town and Arba Minch Zuria woreda. Verbal informed consent from each study participant was obtained after clear explanation about the purpose of the study.

## 3. Results

### 3.1. Sociodemographic Characteristics

Of the 700 respondents included in the analysis, 382 (54.6%) were in the 26–35 age group. The mean age was 31 years with standard deviation (SD) of 6.57 years. Sixty-five percent of the participants were rural, while the rest were urban. According to the study participants score on the six questions used to measure the level of male involvement PMTCT 53% of them were found to be involved by having a score of 4−6 out of the six questions (Table 1).

### 3.2. Level of Male Involvement in PMTCT Program

The level of male involvement in PMTCT was assessed using the variables shown in Table 2. Three hundred fifty-nine (51.3%) of the 700 men had attended ANC with their partners, but most of them 685 (97.9%) out of 700 provided financial support to their spouses to attend ANC. The majority of respondents 366 (52.3%) out of 700 were not willing to use condoms during sexual intercourse for PMTCT of HIV.

### 3.3. Determinants of Male Involvement in the PMTCT Program

In order to measure the association of male involvement in PMTCT with a number of explanatory variables, crude and adjusted odds ratio (OR) with 95% CI were employed and the result is given in Table 3. Compared to those who do not have education, those with education level of above grade 12 (OR (95% CI) = 3.53 (1.29, 9.67)) were more likely to be involved in PMTCT. Non-khat users (OR (95% CI) = 2.71 (1.41, 5.21)) and nonsmokers (OR (95% CI) = 7.29 (1.21, 43.81)) were more likely to be involved in PMTCT than their counterparts (Table 3).

## 4. Discussion

In this study, conducted to establish determinants of male involvement in the PMTCT program in southern Ethiopia, we found that 53% of male partners were involved in the PMTCT program. This level of involvement is higher than what is reported from other studies from East Africa [10]. One study in Kampala, Uganda, showed that male participation in the PMTCT activities was low (16%) [11]. Similarly, a study conducted at a Nairobi antenatal clinic, Kenya, revealed that male partner participation in antenatal VCT with their spouses was low (15%) [12]. This difference can be explained by the difference in background of the study participants and the time gap as better attention has been given to male involvement in PMTCT these days.

In this study we have found a number of factors associated with male participation in the PMTCT program. These included age, residence, education level, knowledge on HIV, knowledge on PMTCT, geographical accessibility of health facility, having weak perception for male involvement in PMTCT, having perception of ANC attendance being females' responsibility, ever use of khat, and ever use of cigarette.

Male partners with comprehensive knowledge on HIV/AIDS were found to be 1.97 times more likely to be involved in the PMTCT program than those who do not have comprehensive knowledge on HIV/AIDS and this could be due to the fact that male partners who do not have comprehensive knowledge on HIV may fail to appreciate the importance of male involvement for prevention HIV infection from mother to child or may have less access to sexual and reproductive health education and promotion in general.

Regarding the association of education level with male involvement in PMTCT in this study, those who have education level of above grade 12 were almost four times more likely to be involved in PMTCT than those who do not have education level. This is in-line with the fact that people that are more knowledgeable could take care of HIV infection, as they easily understood both the transmission and prevention methods. Similar studies in Uganda and elsewhere have found that education level is an important determinant of participation in PMTCT services [11, 13].

In our study men who had knowledge about PMTCT program were almost 2 times more likely to get involved in PMTCT activities than those who had no knowledge. This is consistent with results of a similar study in Tanzania [2]. In addition, another study in Tanzania revealed that males were not fully participating in PMTCT programs and reasons given were lack of information and lack of a direct link between PMTCT staff and males [6].

Cultural factors were also found to be hindering male involvement in the PMTCT program in Arba Minch town and Zuria woreda. For example, antenatal care was perceived a women's affair and having weak perception for males who get involved in PMTCT. In this study compared to those who have weak perception for male involvement in PMTCT, those who do not were almost four times more likely to get involved in PMTCT and those who do not perceive antenatal care as women's affair were two times more likely to get involved in PMTCT than their counterparts. As has been shown in other studies it is conventional in many African cultures for men not to accompany their partners to antenatal and postnatal care consultations as pregnancy and child birth are regarded as women's affair [1].

Geographical accessibility of health facility (within 5 km distance) was positively associated with getting involved in the PMTCT program. The possible explanation for this association could be that the less a health facility is far away from the male partner's house the more a man comes in contact with the health facility with his partner and the more likely he is to get involved in PMTCT. Improving access to ANC is therefore a high priority for improving male involvement in PMTCT of HIV as shown in Thailand [14].

This study has also shown an association between age and male involvement in PMTCT in that those who are in the age range of 36–55 were almost two times more likely to get involved in PMTCT program than those who are 17–25 years old and this might be due to better understanding of older men on PMTCT than their counterparts.

## 5. Limitations of the Study

Being a cross-sectional survey, causality cannot be inferred from these findings and self-report might have also introduced social desirability bias. The data based on Self-declaration of men without women confirmation may limit our result on the evaluation of male involvement in PMTCT. Despite this limitation, the study provides useful information on perception of male partners for involvement of PMTCT that will inform health service planners to design a strategy to increase male involvement in PMTCT of HIV in Ethiopia.

## 6. Conclusion 

The level of male involvement in PMTCT program in Arba Minch town and Zuria woreda was 53% in 2013. Several factors appear to contribute to male involvement in the PMTCT program including age, residence, education level, knowledge on HIV, knowledge on PMTCT, accessibility of health facility, having weak perception for male involvement in PMTCT, having perception of ANC attendance being females' responsibility, ever use of khat, and ever use of cigarette. Geographical accessibility of health facility and male's knowledge on PMTCT should be improved to increase their involvement in PMTCT.

## Figures and Tables

**Table 1:** Sociodemographic characteristics of the study participants in Arba Minch town and Zuria woreda southern Ethiopia, 2014.

Variables	Involved in PMTCT		Total: *n* (%)
	Yes: *n* (%)	No: *n* (%)	
Age [mean (±SD) = 30.77 (±6.57)]			
17–25	79 (11.3)	89 (12.7)	168 (24.0)
26–35	225 (32.1)	157 (22.4)	382 (54.6)
36–55	67 (9.6)	83 (11.9)	150 (21.4)
Residence			
Urban	168 (24.0)	75 (10.7)	243 (34.7)
Rural	203 (29.0)	254 (36.3)	457 (65.3)
Ethnic group			
Gamo	277 (39.6)	270 (38.6)	547 (78.1)
Gofa	9 (1.3)	10 (1.4)	19 (2.7)
Wolayta	45 (6.4)	41 (5.9)	86 (12.3)
Amhara	16 (2.3)	4 (0.6)	20 (2.9)
Others^*∗*^	24 (3.4)	4 (0.6)	28 (4.0)
Religion			
Orthodox Christian	128 (18.3)	93 (13.3)	221 (31.6)
Muslim	18 (2.6)	7 (1.0)	25 (3.6)
Protestant	223 (31.9)	217 (31.0)	440 (62.9)
Others^*∗∗*^	2 (0.3)	12 (1.7)	14 (2.0)
Education			
No education	27 (3.9)	90 (12.9)	117 (16.4)
Primary education	172 (24.6)	152 (21.8)	324 (46.3)
Secondary & above	172 (24.6)	87 (12.4)	259 (37.0)
Occupation			
Government employed	42 (6.0)	16 (2.3)	58 (8.3)
Merchant	60 (8.6)	33 (4.7)	93 (13.3)
Daily laborer	124 (17.7)	120 (17.1)	244 (34.9)
Farmer	92 (13.1)	116 (16.6)	208 (29.7)
Student	53 (7.6)	44 (6.3)	97 (13.9)
Monthly income			
≤300 birr	124 (17.7)	163 (23.3)	287 (41.1)
301–725 birr	130 (18.6)	109 (15.6)	239 (34.2)
≥726 birr	116 (16.6)	57 (8.2)	173 (24.7)

^*∗*^Wolayta, Konso, Gurage, and Oromo; ^*∗∗*^Catholic, Adventist, only Jesus.

**Table 2:** Level of involvement of the study participants in ANC activities in Arba Minch town and Zuria woreda, southern Ethiopia, 2014.

Variables	Respondent's response	
	Yes: *n* (%)	No: *n* (%)
Ever attended ANC with partner	359 (51.3)	341 (48.7)
Knows partner's ANC appointments	415 (59.3)	283 (40.4)
Provides financial support to partner to attend ANC	685 (97.9)	15 (2.1)
Discusses with partner HIV testing	409 (58.4)	291 (41.6)
Do you accept if health professionals inform you to use condom during the time of your wife's pregnancy	334 (47.7)	366 (52.3)
Ever tested for HIV in ANC clinic	375 (53.6)	325 (46.4)
Involved in PMTCT (having score of 4–6)	371 (53)	
Not involved in MPTCT (having score of 0–3)	329 (47)	

**Table 3:** Factors associated with male involvement in PMTCT of HIV among male partners (crude and adjusted OR) in Arba Minch town and Zuria woreda, southern Ethiopia, 2014.

Explanatory variable	Involved in PMTCT		Crude OR (95% CI)	Adjusted OR (95% CI)	*P* value
	Yes (1)	No (0)			
Age					<0.001
17–25	79	89	1.00	1.00	
26–35	225	157	0.62 (0.43, 0.89)	0.51 (0.31, 0.85)	
36–55	67	83	1.10 (0.71, 1.71)	2.07 (1.13, 3.81)	
Residence					
Rural	168	75	1.00	1.00	
Urban	203	254	2.80 (2.02, 3.90)	2.05 (1.31, 3.22)	0.002
Education level					0.021
No education	35	12	1.00	1.00	
Grades 1–4	39	34	2.54 (1.14, 5.66)	1.10 (0.39, 3.12)	
Grades 5–8	133	118	2.59 (1.28, 5.22)	1.36 (0.56, 3.33)	
Grades 9–12	137	75	1.60 (0.78, 3.26)	1.25 (0.50, 3.10)	
>Grades 12	27	90	9.72 (4.44, 21.30)	3.53 (1.29, 9.67)	
Knowledge on HIV					
Yes	122	193	2.89 (2.13, 3.94)	1.97 (1.31, 2.97)	0.001
No	249	136	1.00	1.00	
Knowledge on PMTCT					
Yes	97	156	2.55 (1.86, 3.50)	2.18 (1.39, 3.41)	0.001
No	274	173	1.00	1.00	
Knowledge on ANC services					
Yes	45	238	18.83 (12.69, 27.94)	5.73 (3.64, 9.02)	<0.001
No	324	91	1.00	1.00	
Accessibility of health facility					
Yes	57	98	2.34 (1.62, 3.38)	1.54 (0.94, 2.55)	0.09
No	314	231	1.00	1.00	
Weak perception for male involvement in PMTCT					
Yes	340	223	1.00	1.00	
No	30	106	5.39 (3.47, 8.36)	3.49 (1.82, 6.68)	<0.001
Attending ANC is females' responsibility					
Yes	323	179	1.00	1.00	
No	48	150	5.64 (3.88, 8.19)	1.96 (1.11, 3.45)	0.02
Ever use of khat					
Yes	340	284	1.00	1.00	
No	31	45	1.74 (1.07, 2.82)	2.71 (1.41, 5.21)	0.003
Ever use of cigarette					
Yes	369	312	1.00	1.00	
No	2	17	10.05 (2.30, 43.85)	7.29 (1.21, 43.81)	0.03
